# Barriers and facilitators for individualized rehabilitation during breast cancer treatment – a focus group study exploring health care professionals’ experiences

**DOI:** 10.1186/s12913-020-05107-7

**Published:** 2020-03-26

**Authors:** Ulrika Olsson Möller, Ing-Marie Olsson, Katarina Sjövall, Ingela Beck, Lisa Rydén, Marlene Malmström

**Affiliations:** 1 Department of Nursing and Integrated Health Sciences, Faculty of Health Sciences, Kristianstad University, Kristianstad, Sweden; 2 Department of Health Sciences, Lund University, Box 157, 221 00 Lund, Sweden; 3 Skåne University Hospital, Lund, Sweden; 4 Department of Clinical Sciences in Lund, Oncology and Pathology, Lund University, Lund, Sweden; 5 The Institute for Palliative Care, Lund University and Region Skåne, Lund, Sweden; 6 Lund University, Skåne University Hospital, Department of Clinical Sciences Lund, Surgery, Lund, Sweden

**Keywords:** Breast cancer, Health care professionals, Individualization, Qualitative, Rehabilitation, Barriers and facilitators

## Abstract

**Background:**

Breast cancer (BC) and related treatment are associated with the risk of developing a wide range of persistent disabling impairments. Despite extensive research in the field and an enhanced focus on BC rehabilitation, up to 34–43% of these patients are at risk of developing chronic distress. In addition, it is known that these patients repeatedly report unmet needs, which are strongly associated with reduced quality of life. However, despite knowledge that patients’ needs for support during BC rehabilitation varies greatly, individualized rehabilitation is often lacking. Therefore, this study aimed to explore health care professionals’ (HCPs) experiences of current rehabilitation practice and describe current barriers and facilitators for individualized rehabilitation for patients following BC treatment.

**Methods:**

A total of 19 HCPs were included, representing various professions in BC care/rehabilitation within surgical, oncological and specialized cancer rehabilitation units at a university hospital in Sweden. Five semi structured focus group interviews were conducted and inductively analysed using conventional qualitative content analysis.

**Results:**

Three categories were captured: (1) varying attitudes towards rehabilitation; (2) incongruence in how to identify and meet rehabilitation needs and (3) suboptimal collaboration during cancer treatment. The results showed a lack of consensus in how to optimize individualized rehabilitation. It also illuminated facilitators for individualized rehabilitation in terms of extensive competence related to long-term experience of working with patients with BC care/rehabilitation. Further, the analysis exposed barriers such as a great complexity in promoting individualized rehabilitation in a medically and treatment-driven health care system, which lacked structure and knowledge, and overarching collaboration for rehabilitation.

**Conclusion:**

This study suggests that the cancer trajectory is medically and treatment-driven and that rehabilitation plays a marginal role in today’s BC trajectory. It also reveals that structures for systematic screening for needs, evidence-based guidelines for individualized rehabilitation interventions and structures for referring patients for advanced rehabilitation are lacking. To enable optimal and individualized recovery for BC patients’, rehabilitation needs to be an integrated part of the cancer trajectory and run in parallel with diagnostics and treatment.

## Background

Despite a favourable prognosis and extensive evidence of the positive effects of cancer rehabilitation, patients with breast cancer (BC) still suffer from unmet rehabilitation needs [1]. This may increase the risk of prolonged or inhibited recovery, emphasizing the need for extended knowledge about barriers and facilitators for individualized rehabilitation.

BC is the most common cancer in women worldwide and is responsible for about 28% of all cancer diagnoses [2]. Diagnostic and treatment related advances have resulted in decreased mortality [3] and prolonged survival [4, 5]. Despite favourable survival outcomes, BC and associated treatment unfortunately comes with the risk of developing a wide range of persistent disabling complications. Studies show that 34–43% of patients with newly diagnosed BC report high distress [6, 7] and therefore are at risk of developing chronic distress [7] and more than 60% report at least one adverse treatment effect 6-year after diagnosis [8]. Distress is defined as a multi-factorial, unpleasant emotional experience of a psychological, social and/or spiritual nature that may interfere with the ability to cope effectively with cancer and its physical symptoms and treatments [9]. As BC also tends to be diagnosed at younger ages compared with other common cancer types [4] the long-term impact also affects work ability, with approximately 30–60% remaining on sickness absence 1 year after treatment [10, 11].

This means that rehabilitation is essential for patients’ suffering from BC as cancer rehabilitation aims to prevent and reduce the physical, psychological, social and existential consequences of cancer [12]. Studies have repeatedly shown that exercise and physical activity have positive effects on several consequences of BC treatment such as reduced fatigue, depression, anxiety, and lymphoedema as well as increased shoulder mobility and Quality of Life (QoL) [13–16]. However, studies have also reported that BC patients have unmet rehabilitation needs in relation to fear of cancer recurrence, psychological concerns, having someone to talk to [17], patient-education and psychological, financial and occupational counselling [1]. Unmet needs are strongely associated with decreased QoL [18]. Parallel to this a recent systematic review showed that one symptom or problem could be treated with a wide range of interventions and that the efficacy depend on the diverse array of aetiological causes underlying the problem, and patients’ diverse preferences [19]. Altogether this indicate that individualization is essential for optimized recovery.

For cancer rehabilitation to be successful, teamwork is needed to prevent and reduce the physical, psychological, social and existential consequences of cancer and its treatment [12]. In Sweden, national guidelines for cancer rehabilitation [20] include recommendations on assessment: care processes, care structure and teamwork: treatments: self-care: physical, psychological, social and existential aspects: follow-up and quality indicators. However, implemetention and clinical effects of the national cancer rehabilitation guidelines has not yet been investgated.

Despite extensive research related to BC rehabilitation, the reasons behind patients’ unmet rehabilitation needs are still unclear. Therefore, interviewing HCPs about barriers and facilitators for individualized rehabilitation is needed to gain a deeper understanding of reasons for patients’ lack of access to rehabilitation.

## Methods

The aim of this study was to explore HCPs’ experiences of current rehabilitation practice and describe current barriers and facilitators for individualized rehabilitation for patients following BC treatment. This explorative qualitative focus group study is a part of the ReScreen complex intervention study (Clinicaltrials.gov NCT03434717) focusing on screening-based individualized rehabilitation following BC treatment. The overall project is developed according to the Medical Research Councils (MRC) framework for complex interventions [21]. The present study is a part of the first phase of the complex intervention framework “Development” focusing on identifying the evidence base. These results combined with the results of a systematic review of reviews [19] will be used as a fundament in the development of an intervention for evidence-based rehabilitation which is evaluated in the third phase of the project. The manuscript is reported according to the Consolidated criteria for reporting qualitative research (COREQ) guidelines.

### Context

This study was conducted at the Departments of Surgery and Oncology of a university hospital in southern Sweden where approximately 670 patients are diagnosed with BC annually. In Sweden, rehabilitation in this context, is performed at different levels depending on available competence and resources. Patients with BC are depending on initial treatment regime treated and followed-up at the surgical or at the oncological outpatient unit. These outpatient units represent the basic rehabilitation level. At the basic rehabilitation level contact nurses working specifically with BC patients are the patients primary care contact and are available during the pre and post treatment phase. These units also include rehabilitation resources such as physiotherapists, occupational therapists and social workers. Some rehabilitation follow-ups are structured, for example all patients with axillary lymph node dissection, by default see a physiotherapist before and after surgery focusing on lymphedema prevention, while rehabilitation in general is based on patients’ initiatives. Patients that, at the basic rehabilitation level, are identified as having complex needs can be referred to a specialized cancer rehabilitation unit (hereafter referred to as the advanced rehabilitation level). The unit for advanced rehabilitation is organized under the Department of Oncology and include a multi professional team with e.g. physicians, psychologist, social workers, physiotherapists and occupational therapists that exclusively focus on rehabilitation of patients with cancer.

### Recruitment and participants

Different HCPs working within BC care or specific cancer rehabilitation, at the Departments of Surgery and Oncology and in various parts of the cancer trajectory, were considered eligible for inclusion. Participants were purposefully included through key persons, by mail or face-to face, at each unit to achieve maximum variation regarding type of profession, workplace, and years of working with BC/in cancer rehabilitation. A total of 19 HCPs from the Departments of Surgery (*n* = 11) and Oncology (*n* = 8) were included representing nurses (*n* = 7), nurse assistants (*n* = 1), physicians (*n* = 1), psychologists (*n* = 1), physiotherapists (*n* = 5), social workers (*n* = 3) and occupational therapists (*n* = 1). Four of the participants had worked in the field for 1–5 years, three for 6–10 years, five for 11–20 years, three for 21–30 years and four for 30 years or more.

### Focus group interviews

Five focus group interviews [22] with three to five participants in each group were conducted in November 2016 – March 2017. The interviews lasted between 71 and 89 min and were conducted in a conference room at the hospital. A semi-structured interview guide was used (see Additional file 1), focusing on HCPs’ experiences of current rehabilitation practice and barriers and facilitators for individualized rehabilitation. The interviews started with an open-ended question: “Could you please describe your role in the rehabilitation of patients following BC treatment?” which was followed by probing questions to get a deeper understanding and illuminate various perspectives. The last author (M.M) moderated the interviews aiming to support the participants in focusing on the study aim while one assistant interviewer (I.B, U.OM, or K.S) kept notes and asked probing questions [22]. To validate the interpretation of the interviews an assistant interviewer summarized the content of the interviews at the end of each interview, thereby allowing for immediate member checking [23]. When conducting the fifth interview no new information emerged why data collection was closed.

### Data analysis

The digitally recorded and transcribed interviews were analyzed using conventional qualitative content analysis [24]. This inductive approach allows categories to flow from the data. The first and last authors (U.OM, M.M) had the main responsibility for the analysis while the other authors focused on ensuring the link between the data and the analysis. All researchers have extended experiences of qualitative research and focus group interviews. Initially, all transcripts were read and/or listened to repeatedly by the authors independently to achieve an overall understanding and a sense of the whole. Thereafter, words and meaning units in the text that highlighted key concepts were identified independently, and notes were made about the initial analysis. An initial coding scheme was developed by defining and labelling patterns, sub-categories and categories. Similarities and differences in the interpretation of data were discussed throughout the analysis until consensus was reached by all authors.

## Results

The participants’ experiences were captured in three categories and eight sub-categories (Table 1) describing current rehabilitation practice as well as barriers and facilitators for individualized rehabilitation. Extensive professional competence, related to experiences of working with patients with BC/rehabilitation, was described as a facilitator for individualized rehabilitation as it enabled experience-based identification of patients’ needs. Barriers for individualized rehabilitation, on the other hand*,* were identified in terms of lack of structure, collaboration and knowledge in relation to individualized rehabilitation in an often medically and treatment-driven health care system.

**Table 1:** Schematic overview of categories and sub-categories

Varying attitudes towards rehabilitation	Incongruence in how to identify and meet rehabilitation needs	Suboptimal collaboration during cancer treatment
Rehabilitation based on medical indicators	Identifying signs of vulnerability	Interprofessional team collaboration
Lack of consensus about approach towards rehabilitation	Screening for rehabilitation needs	Interdisciplinary collaboration
Setting goals for rehabilitation	Actions triggered by signs of vulnerability	

### Varying attitudes towards rehabilitation

The participants’ attitudes towards rehabilitation could be a barrier to patients’ access to rehabilitation. This was apparent when rehabilitation and follow-up were based on medical indicators, such as side effects of various treatments, rather than on patients’ individual needs and when consensus about the goal of rehabilitation was lacking.

### Rehabilitation based on medical indicators

Rehabilitation, in the context of follow-up, were on the basic level predominately described as organized based on medical indicators such as risk of developing lymphoedema or risk of side effects associated with specific treatments. However, the participants stressed that the assessments of patients’ rehabilitation needs should be based on a combination of personal characteristics, such as the patient’s social network, personality and life situation. Therefore, the importance of exploring the patient’s life situation and resources at an early stage was repeatedly emphasized. At the advanced cancer rehabilitation level, a more comprehensive approach to rehabilitation was adopted, allowing for screening for both needs and resources as a base for a rehabilitation plan.

I use it [screening tool] not just to identify problems but also to capture resources. What support does this person have in her life to cope with her current life situation, and how can we contribute? (Participant at advanced rehabilitation level, Interview 5).

The participants emphasized the importance of preparing patients for potential problems by giving them information in advance e.g. about risk of developing fatigue. They also stressed the importance of empowering patients to call the contact nurse if needed. However, participants also described the risk of patients not knowing when to call as they might be unaware of symptoms and problems that might prompt a contact.

…the health care service has a great responsibility to at least make sure of it [that patients know when to call], because it is very much based on the fact that *they* should contact *us*. Therefore, we have to make sure that they know where to go… (Participant at basic rehabilitation level, Interview 1)

### Lack of consensus about approach towards rehabilitation

The approach towards rehabilitation varied depending on the participants’ individual knowledge about and interest in rehabilitation, and on what rehabilitation level the HCP worked. It was evident that patients could be given different advice and support depending on which HCP they see. This indicate that lack of knowledge and consensus about timing and strategies for rehabilitation is a barrier. The HCPs expressed diverse views on the best time to initiate a rehabilitation plan. Some participants felt it was insulting to discuss rehabilitation when the patient is facing a life-threatening illness and stressed that patients should take it easy and slowly adjust to the new situation with as little interference from the HCPs as possible.

And the most important thing is not that [rehabilitation]; the important thing is to get them to understand the diagnosis and support them during the treatment. Then, if you feel during these meetings that ‘there seems to be a problem here’ then of course you can document it /…/ but it is not primary, that we need to identify their rehabilitation needs. (Participant at basic rehabilitation level, Interview 3)

On the other hand, it was also emphasized that a proactive approach promoting rehabilitation at an early stage was fundamental to recovery. At the same time HCPs repeatedly described rehabilitation as driven by patients’ initiatives indicating an inactive rather than proactive rehabilitation. Altogether, this indicated a lack of consensus regarding the meaning of and approach towards rehabilitation.

### Setting goals for rehabilitation

Participants sometimes described specific goals, both for rehabilitation in general and for establishing goals for each patient. However, at the basic rehabilitation level goal setting was often not described as a part of clinical routines and were rarely discussed at a team level. Specific patient-related goals were highlighted as important, such as being able to touch the breast or to get back to work but were rarely developed in conjunction with the patient. It was also stressed that there was a need for an integrated individual rehabilitation plan in which the patient’s goals were clearly documented, and which followed the patient throughout the cancer trajectory to optimize rehabilitation.

It [the rehabilitation] starts there in the screening moment /…/ but above all … The approach is always that ‘You are going back [to recover]’, and how do we best achieve this? (Participant at basic rehabilitation level, Interview 3)

### Incongruence in how to identify and meet rehabilitation needs

The importance of identifying patients’ needs throughout the cancer trajectory was stressed. Still, a lack of structure for continuous screening for rehabilitation needs was repeatedly described as a barrier and consensus about the need for systematic needs assessments was lacking. Rather, HCPs identified signs of vulnerability as indicators for rehabilitation needs based on clinical experience.

### Identifying signs of vulnerability

Based on experience, the HCPs were attentive to signs of vulnerability by looking beyond the merely obvious and medical aspects. These signs varied and could include specific patient groups such as younger women with children, women that were alone in their situation or did not seem to understand the situation:

It comes naturally; I can see if a patient has small children or not … or if a patient has a chaotic relationship or if her husband has just left her …// It just something you catch in the moment … (Participant at basic rehabilitation level, Interview 2)

By identifying signs of vulnerability, the participants extended the understanding of patients’ needs. For example, if a patient had a history of burn-out the HCPs would ask how this might influence her rehabilitation or, in women with children, to explore the plans for telling their children about the cancer. However, the participants also described the risk of potential signs being neglected if they were considered “sensitive” (such as relational, sexual or existential issues) or if the HCP was unaware of specific signs.

The participants described that the contact nurses at the basic levels were best positioned to identify signs of vulnerability. The other HCPs corroborated this, saying they had faith in the contact nurses’ ability to identify and refer patients in need of their interventions.

I think teamwork is everything in this, because you [the nurses] see it [the needs], you are the ones who meet the patient first. And then the rest of us come in. (Participant at basic rehabilitation level, Interview 3)

The participants highlighted that patients’ needs vary greatly and that HCPs need to be aware of and responsive to patients’ needs throughout the cancer trajectory, which was described as a complex task.

### Screening for rehabilitation needs

Even if the participants at the basic level mainly described similar potential signs of vulnerability there were no consensus about or structure for how or when these potential signs should be identified. Various opinions were expressed about the importance of adopting a more structured way of screening. Some participants expressed that the most important issue was the relational aspects of support; they said that a more structured approach towards rehabilitation (e.g. through screening) could compromise the patient–HCP relationship, as the approach became instrumental and objective.

Some HCPs like to have a sheet and be able to count and measure, while other colleagues believe that /… / it should come naturally and that establishing a relationship is needed for concerns to emerge. (Participant at advanced rehabilitation level, Interview 5)

Others stated that there was a great need for an improved and reliable way of identifying patients’ needs. These participants argued that a structured screening procedure would make it possible to identify and act upon each patient’s needs and would provide better prerequisites for individualized rehabilitation. On the other hand, participants at the advanced level stressed that patients often were referred too late indicating the need of earlier identification of patients’ needs.

We have tried to convey this [earlier needs assessment] //, that this should be done before they [the patients] come to us. // They come, as I see it, too late. (Participant at advanced rehabilitation level, Interview 5)

### Actions triggered by signs of vulnerability

The participants described that when signs of vulnerability were identified and a patient was considered to be in need of extended rehabilitation, this triggered various actions. If the needs were practical (e.g. economic issues), psychological (e.g. anxiety) or physical (e.g. swollen arm) there was usually a clear routine for referral to a social worker or physiotherapist at the basic rehabilitation level. By contrast, if the trigger was based on personal characteristics or health behaviours (e.g. inactivity, substance abuse), a structure for initiating supportive interventions or referring patients for extended support was often lacking.

### Suboptimal collaboration during cancer treatment

It was evident that barriers for rehabilitation existed at an organizational level, in terms of lack of interprofessional (between HCPs at the same department) and interdisciplinary (between the departments) collaboration. This was described as hindering a comprehensive rehabilitation process in which a team-based rehabilitation plan follows the patient throughout the cancer trajectory.

### Interprofessional team collaboration

“Interprofessional collaboration” was often referred to as gathered professional competences within a specific unit, meaning that the team collaboration was related to one part of the cancer trajectory. It was stressed that interprofessional discussions about patient cases were important to keep updated, to enhance knowledge and to ensure that rehabilitation recommendations were valid, and evidence based. Still, at the basic rehabilitation level, the structure for team collaboration was limited. To enhance such collaboration, geographic and resource aspects were highlighted as important. For example, working geographically close made it easier to discuss individual patients and to share experiences in an informal way while the HCPs working in several units often described their role as consultative.

There is probably no one, I believe, who thinks that it [team-based evaluations] would be impossible, but there could be purely practical conditions, obstacles, organizational barriers for it to happen spontaneously. //. But right now, there is no structure for it … // You make the best out of what you have. (Participant at basic rehabilitation level, Interview 1)

### Interdisciplinary collaboration

Interdisciplinary collaboration was often described as unsatisfactory. The ineffective collaboration was described as a process where the next instance “started over instead of taking over”, leading to a potential risk of prolonged waiting times, inefficient communication, and sub-optimal rehabilitation. To enable optimal rehabilitation, it was stressed that the recovery period should be (but often was not) seen from a comprehensive continuous perspective.

Rehabilitation should really run through it all. From day one when you get your diagnosis, until you return to your normal everyday life. (Participant at advanced level, Interview 4)

One example of the lack of collaboration between disciplines was that despite extensive available competence at the advanced rehabilitation level many participants at the basic level was either unaware of the unit’s existence or of how or when to refer patients. The participants also expressed that they were working in a quickly changing health care system within an area where new evidence rapidly emerged, which made it difficult to keep up to date with both evidence and available rehabilitation resources. Therefore, they expressed that there was a need for a support tool that included information about which interventions might be effective for different problems and information about locally available rehabilitation resources.

There is not much to offer … who should we refer them to? (Participant at basic rehabilitation level, Interview 3)

Therefore, implementation of evidence-based guidelines that are adopted throughout the cancer trajectory was expressed as having potential to bridge the gap between different disciplines.

## Discussion

Despite national guidelines and initiatives to integrate rehabilitation throughout the cancer trajectory, this study shows that rehabilitation plays a marginal role in today’s BC care in Sweden. The results demonstrate a prominent lack of consensus regarding HCPs approach towards rehabilitation. It also demonstrates that the responsibility for identifying patients with extended rehabilitation needs is on the basic level where structures for systematic needs assessments and evidence-based guidelines often are lacking. This imbalance is likely to be a barrier for individualized rehabilitation. Altogether, these results clearly show that there is a gap between rehabilitation research and clinical practice leaving patients with sub-optimal rehabilitation and emphasizing the need for implementation of guidelines for individualized rehabilitation.

This study shows that rehabilitation often is organized based on medical or treatment-related indicators meaning that patients with more advanced or specific treatments are more actively monitored. This way of organizing rehabilitation and survivorship care has also been demonstrated in a Danish study [25] where cancer patients’ initial care often was described as based on the novelty and severity of the diagnoses, with focus on treatment, side effects and care, which drew attention and focus away from survivorship care. These results are in line with previous research showing that medical indicators, such as type of tumour, are likely to be modest indicators for distress and that poor QoL, disability, or unmet needs are more powerful predictors of distress [26] indicating an extended rehabilitation need. HCPs in the present study stress that patients’ individual preferences and signs of vulnerability are important indicators for patient’s rehabilitation needs, which is in line with former research [25, 26] indicating a medical driven rehabilitation system.

Earlier studies have shown that HCPs avoid structured assessments because of insufficient implementation of a needs assessment form, uncertainty [27], or because they question the added value of screening tools [28, 29]. In contrast, studies also show perceived benefits of assessment of needs including detecting needs, enhancing holistic care, improving clinician–patient relationship and enhanced potential to address problems [27]. These dual perspectives relating to the value of structured evaluations was also seen in the present study where some HCPs expressed that the experienced based and “informal” identification of needs was a facilitator for individualized rehabilitation while others described it as a barrier since it came with a risk of HCPs addressing different problems. This indicates that even if patients are identified they might get varying advice depending on whom they see and where they are in the cancer trajectory. A screening test is, however, usually not enough for facilitating change in patient outcomes. Rather it could be seen as the first step in a process where further comprehensive assessments and timely provision of evidence-based intervention are needed [26]. As stated by Stout et al. [30], describing the Prospective Surveillance Model (PMS), patient’s self-identification of rehabilitation needs is insufficient, which is also the base for the present study. However, instead of focusing primarily on physical and functional limitations, as in the PSM, the present study adds knowledge of barriers and facilitators from the perspective of different HCPs and for early identification of physical, psychological, existential and/or social needs that would trigger automatic referral. The results from the present study shows that HCPs’ stressed the need for an evidence-based decision support tool where the latest evidence is combined with locally available rehabilitation resources as a basis for ensuring evidence-based individualized rehabilitation.

Identifying women with extensive rehabilitation needs is a complex but fundamental task when aiming to ensure optimized BC rehabilitation. In this study, specialist and profession-specific competence were highlighted as important for enabling individualized rehabilitation. It was clearly stated that the responsibility for identifying women with extended needs was dependent on the contact nurses on the basic rehabilitation level. However, it was unclear who had the overall responsibility for the rehabilitation process, and an overall plan and structure for when and how patients should be referred within the system was lacking. Studies stress the importance of a multidisciplinary approach to meet the wide range of rehabilitation needs of patients with BC [31, 32] which also was emphasised in the present study. Despite this, interprofessional collaboration was described as insufficient in terms of “the next instance is starting over instead of taking over”. The lack of collaboration resulted in limited knowledge about resources between disciplines and can be seen as a sign of a fragmented cancer care trajectory.

This study shows that despite an extensive amount of research within the field structures for individualized rehabilitation is lacking. It is also well known that research often fail to translate into meaningful patient care outcomes. Key domains that are fundamental to consider to successfully implement research findings into clinical practice include understanding barriers and facilitators related to the characteristics of the intervention and the involved individuals, the inner and outer setting and the process of implementation [33]. This emphasize the need for a deeper understanding of barriers for implementing individualised rehabilitation to provide a solid evidence ground for further development within this field. In the present study barriers related to individuals’ attitudes towards rehabilitation, planning and organizing rehabilitation and lack of inter disciplinary collaboration were revealed. These different perspectives need to be considered in the further development towards individualized rehabilitation for patients suffering from BC.

### Strengths and limitations

This qualitative focus group study is, to our knowledge, the first of its kind exploring HCPs’ experiences of current practice and barriers and facilitators for individualized rehabilitation for patients treated for BC. Including HCPs who represent various professions and disciplines allowed for variation in perspectives, which can be considered a strength of this study. The design also enabled a deeper understanding of the mechanisms behind the current routines. However, this also means that narratives from different parts of the trajectory were included which might be related to conditions that are unique to that specific specialty and this should be considered when interpreting the results. When participant have intense or lengthy experience within the topic smaller focus groups are often recommended [22]. In this study we therefore aimed for smaller focus groups to enhance the opportunity to share insights and observations which is considered a strength of the study. The focus group interviews were rich and dynamic reflecting that the participants felt comfortable in talking about this topic. No further information emerged during the fifth interview why data collection was closed. The included participants reflect the current composition of HCPs working within cancer rehabilitation. The fact that only one physician and one psychologist were included reflects this composition but might be considered as a limitation of the study. A further consideration is that the study exclusively includes representatives from hospital-based cancer rehabilitation which might be a potential limitation since the primary health care (community based) perspective is lacking. Further studies focusing on cancer rehabilitation in the transition between hospital-based and primary health care is therefore needed.

To increase the trustworthiness of the study [34] the HCPs were encouraged to share their experiences without any pressure to reach consensus. Each interview was moderated and conducted by researchers with experience in conducting qualitative focus group interviews and member checking was used to ensure that the interviews captured the HCPs’ experiences. The last author had a distant professional relationship with some of the participants while the others had no previous relationship. To increase trustworthiness a second researcher without relationship with the participants were present at the interviews.

Transferability of qualitative studies is related to the degree of similarity between the study context and the clinical setting. Authors may make suggestions about transferability but due to contextual differences it is always the readers’ responsibility to determine however or not it is transferable [34]. This study has limitations related to being performed at a single center and being performed within the Sweden health care system only. However, since a qualitative study do not aim to generalize rather to describe variations, we suggest that these results are likely to mirror the role of cancer rehabilitation in similar cancer settings where evidence-based rehabilitation guidelines may not yet have been implemented.

## Conclusion

Despite the participants’ extensive individual knowledge in the field, this study clearly shows that the cancer trajectory is medically and treatment-driven and that rehabilitation plays a marginal role in the earlier parts of the trajectory. It also demonstrates a prominent imbalance in the role and structure of rehabilitation as the responsibility for identifying patients with extended rehabilitation needs is at the basic level where rehabilitation is often not an integral part. Structures for systematic screening for needs, evidence-based guidelines for individualized rehabilitation interventions and structures for referring patients for advanced rehabilitation are often lacking. To enable optimal and individualized recovery for BC patients’, rehabilitation needs to be an integrated part of the cancer trajectory and run in parallel with diagnostics and treatment.

## Supplementary information

**Additional file 1.** Interview guide.

## Data Availability

This study has in accordance with the national ethical regulations (2003:460) received ethical approval from the Regional Ethical Review board in Lund, Sweden. In the approved application (reference number 2015/505) we stated that only the members of the research group that are involved in the study will have access to data. This means that we are not allowed to share the raw data from this study.

## Abbreviations

BC: Breast cancer
HCP: Health care professional
QoL: Quality of Life
