# Supplementary material for: Barriers and facilitators for individualized rehabilitation during breast cancer treatment – a focus group study exploring health care professionals’ experiences
Source: BMC Health Serv Res. 2020 Mar 26;20:252. doi: 10.1186/s12913-020-05107-7 (PMC7098158; doi:10.1186/s12913-020-05107-7)
Supplement: Supplementary file 1 — Additional file 1. Interview guide. [file 12913_2020_5107_MOESM1_ESM.docx]

**INTERVIEW GUIDE**

**the ReScreen study**

**Could you please describe your role in the rehabilitation of patients following BC treatment?**

Please describe and discuss:

- the rehabilitation process on your clinic as well as external actors
- factors that affect patient’s rehabilitation needs
- the basis (clinical, scientific) for introducing/recommending various rehabilitation interventions
- the follow-up procedure of your patients
- your team collaboration
- yours versus patient’s responsibility for their rehabilitation
- how to motivate patients for rehabilitation
- your resources for rehabilitation
- barriers and facilitators for rehabilitation
- optimal rehabilitation following BC treatment

Contact: [marlene.malmstrom@med.lu.se](mailto:marlene.malmstrom@med.lu.se)
